# Structural and Energetic Evidence Supports the Non-Covalent Phosphate Cyclization by the Class II Phospholipase D from *Loxosceles intermedia*

**DOI:** 10.3390/toxins17030111

**Published:** 2025-02-27

**Authors:** Carolina Gismene, José Fernando Ruggiero Bachega, Daniel Z. Doherty, Silvio Sanches Veiga, Raghuvir K. Arni, Jorge Enrique Hernández González

**Affiliations:** 1 Biological Structures Group, Multiuser Center for Biomolecular Innovation (CMIB), São Paulo State University—UNESP, São José do Rio Preto CEP 15054-000, SP, Brazil; carolina.gismene@unesp.br (C.G.); daniel.z.doherty@adelaide.edu.au (D.Z.D.); raghuvir.arni@unesp.br (R.K.A.); 2 Graduate Program in Molecular and Cellular Biology, Federal University of Rio Grande do Sul (UFRGS), Porto Alegre CEP 90050-170, RS, Brazil; ferbachega@ufcspa.edu.br; 3 Departament of Farmacosciences, Federal University of Health Sciences of Porto Alegre (UFCSPA), Porto Ale-gre, CEP 90050-170, RS, Brazil; 4 Department of Cell Biology, Federal University of Paraná (UFPR), Curitiba CEP 81531-980, PR, Brazil; veigass@ufpr.br

**Keywords:** phospholipase D, *Loxosceles*, spider venom, crystal structure, cyclic phosphate, catalytic mechanism

## Abstract

Phospholipase D (PLD) enzymes from *Loxosceles* spider venom mediate envenomation pathology by cleaving phospholipid headgroups. We revisited the crystal structure of *Loxosceles intermedia* PLD (PDB: 3RLH) to evaluate two alternative mechanisms—covalent and non-covalent—for headgroup cleavage. The covalent mechanism involves a nucleophilic attack on the substrate’s P atom by catalytic histidine, forming a phosphohistidine intermediate. It was originally suggested that this intermediate hydrolyzes, leading to linear phosphates. The non-covalent mechanism relies on the substrate’s hydroxyl group performing an intramolecular attack on the P atom, thereby generating a cyclic phosphate. Structural refinement of the crystal structure revealed a cyclic phosphate bound at the active site, replacing previously assigned PEG molecules. This cyclic product, stabilized by His12, His47, and Mg^2+^, provides structural evidence that supports phosphate cyclization. The results of computational analyses, including molecular dynamics and quantum mechanics/molecular mechanics simulations, further support the non-covalent mechanism as the energetically preferred pathway, with a significantly lower activation barrier. Our findings highlight the role of substrate orientation and of the catalytic His residues in transphosphatidylation, advancing our understanding of PLD enzymology and providing insights for the design of inhibitors against *Loxosceles* envenomation.

## 1. Introduction

Phospholipases D catalyze the cleavage of the polar headgroup from phospholipids [1]. They are also implicated in triggering and regulating a wide spectrum of crucial cellular processes, including adhesion, endocytosis, vesicle transport, cell migration, exocytosis, and cell death [1,2]. PLDs enzymes have been identified in the venom of *Loxosceles* spiders (commonly known as brown spiders), where they are believed to play a key role in the venom’s toxic effects [1]. In mammals, spider venom PLDs may lead to complement-dependent dermonecrosis, neutrophil recruitment, and the onset of cutaneous loxoscelism, [3,4] which, in the case of *Loxosceles laeta*, has been demonstrated to increase the expression/secretion of matrix metalloproteinases (MMPs) [5]. Spider venom PLDs also exhibit systemic effects and intravascular hemolysis and may cause acute renal failure, which can result in death [6]. PLDs identified in non-venomous spider species are primarily employed for prey digestion and degradation [7]. Interestingly, homologs have also been identified in various pathogenic microorganisms [8], e.g., *Corynebacterium pseudotuberculosis* [9,10], *Arcanobacterium haemolyticum* [2,11], and *Austwickia chelonae* [12].

PLDs possess molecular weights ranging from 17 to 220 kDa and share limited sequence homology across different species [1,13]. Despite their structural differences, they produce similar pathophysiological effects. These enzymes are thought to have evolved from the structurally related glycerophosphoryl diester phosphodiesterases (GDPD, EC 3.1.4.46), which hydrolyze glycerol phosphodiesters and play a key role in glycerol metabolism [14,15].

Early studies demonstrated the activity of spider venom PLDs against sphingomyelin (SM), hence their usual designation as sphingomyelinases (SMases) [16,17,18]. Interestingly, subsequent experiments indicated a broader substrate specificity for these enzymes, including other ceramide-based phospholipids, e.g., ceramide phosphoethanolamine (CPE), and lysophospholipids, e.g., lysophosphatidylcholine (LPC) and lysophosphatidylethanolamine (LPE) [19]. One of the products of the reaction catalyzed by spider venom PLDs is the lipid substrate’s polar headgroups, which are primarily choline and ethanolamine. The second product formed was generally assumed to be either ceramide-1-phosphate (C1P) or lysophosphatidic acid (LPA), depending on whether the cleaved substrate was a ceramide-based phospholipid or a lysophospholipid, respectively [17,20,21,22,23,24]. Murakami et al. proposed a covalent mechanism for the cleavage of SM by *L. intermedia* PLD, which involves the nucleophilic attack of His12 on the substrate’s P atom [20]. The ability of the catalytic residue His12 to act as a nucleophile was supported by compelling structural evidence obtained for a bacterial PLD [25]. Interestingly, in a later version of the covalent mechanism, the roles of His12 and His47 were swapped, with His47 instead being responsible for the nucleophilic attack on the substrate [14].

More recent studies have focused on the chemical characterization of the products of the phospholipid cleavage catalyzed by spider venom PLDs using ^31^P NMR and mass spectrometry [19,26,27]. These experiments showed that spider venom PLDs exclusively catalyze transphosphatidylation rather than hydrolysis, forming a cyclic six-membered ceramide(1,3)phosphate (CC(1,3)P) and a cyclic phosphatidic acid (CPA) from the two major substrates SM and LPC, respectively [26]. Similar findings were reported for PLD toxins from pathogenic bacteria and fungi [27]. Based on these results, a novel mechanism was proposed, which involves an intramolecular nucleophilic attack by the hydroxyl group at C3 of the phospholipid on its P atom, facilitated by the catalytic histidine residues of PLDs, i.e., His12 and His47 [19,28]. This mechanism will be referred to as ‘non-covalent’ in contrast to the covalent mechanism, which requires the formation of a His-phospholipid adduct [14,28]. Computational studies have provided further evidence supporting the non-covalent mechanism. For example, a structural model for the interaction of SM with an *L. intermedia* PLD, generated by docking and refined through molecular dynamics (MD) simulations, suggested that the conformation of the substrate is optimal for promoting the subsequent cyclization step [28,29]. However, more studies are necessary to elucidate the catalytic mechanism of spider venom PLDs.

Encouraged by the current scenario, we reanalyzed a crystal structure of *L. intermedia* previously deposited in the Protein Data Bank (PDB ID: 3RLH), which evidenced electron density corresponding to unidentified molecules bound at the active site [30]. Our current results indicate that this density corresponds to that of a cyclic phosphate that coordinates the Mg^2+^ ion at the PLD active site. We hypothesized that this molecule was formed through the action of the PLD on molecules present in the purification and crystallization buffers [30], following a series of steps similar to those involving the natural substrates. We also conducted a computational study to analyze the free energy profiles associated with the two proposed mechanisms for the cleavage of polar headgroups by spider venom PLDs. The findings demonstrate that the transition states of the non-covalent mechanism have lower energy compared to those of the covalent mechanism. In addition to the mechanistic insights provided by the previous results, our study also offers valuable information for designing molecules that inhibit the catalytic activity of PLDs.

## 2. Results

### 2.1. Crystallographic Evidence for the Cyclic Phosphate Bound at the PLD Active Site

**Figure 1:**
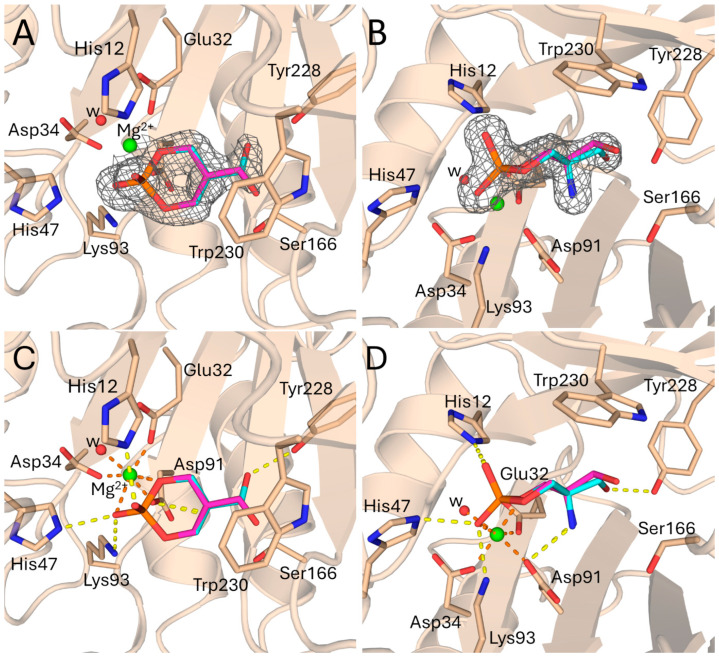
Electron density and interactions at the ligand binding site of PLD from *L. imtermedia* (PDB: 3RLH). (**A**,**B**) Side and overhead views of ACP, modeled in two conformations (cyan and magenta, with occupancies of 60 and 40%, respectively) within the active-site electron density. The protein backbone is presented as a cartoon (wheat-colored). The residues involved in catalysis, metal ion binding, and hydrogen bonding with ACP are highlighted as sticks. The ACP electron density map (grey mesh, σ = 1.0, carve radius = 1.6) is shown surrounding the ACP ligand and was generated from a composite omit map (2mFo-DFc). The magnesium atom (green) and active-site water molecule (red) are presented as spheres. (**C**,**D**) Side and overhead views of the hydrogen bonding and Mg coordination bond interactions with ACP. The hydrogen bonds between the ligand and active-site residues are highlighted as yellow dashed lines, while the coordination bonds with the Mg atom are shown as orange dashed lines.

The replacement of two PEG4 molecules with two conformations of a cyclic phosphate ring containing an amine (-NH_2_) and a hydroxymethyl (-CH_2_OH) group at carbon C2 (Figure 1 and Figure S1, Supporting Information) and refinement resulted in an improved R_free_ (Table 1). The newly positioned ligand at the active site is a known compound with the IUPAC name (5-amino-2-hydroxy-2-oxo-1,3,2λ^5^-dioxaphosphinan-5-yl)methanol (abbreviated as ACP), available in PubChem under CID 10130243. Additionally, during the reprocessing of the *L. intermedia* PLD crystallographic structure, six water molecules were incorporated, while the Mg^2+^ ion and other ligands (PGE, PEG, and EDO) (Figure S1, Supporting Information) were retained [30]. The electron density in the space occupied by the ligand (Figure 1A,B) is highly suggestive of the presence of a six-membered ring, which coordinates in a bidentate fashion to the Mg^2+^ cofactor (Figure 1C,D).

The analysis of the intermolecular hydrogen bond (H-bond) network at the interface revealed key interactions between ACP and active-site residues. Two phosphate oxygens form H-bonds with His12, His47, and Lys93 (Figure 1C,D). At the opposite end of ACP, the hydroxyl oxygen adopts two alternative orientations, resulting from a rotation of ~122° around the C–CH_2_OH bond, with occupancies of 60 and 40%, as determined by the electron density. In the higher-occupancy position, this oxygen interacts with the hydroxyl group of the Tyr228 side chain (Figure 1C,D). Additionally, the amine nitrogen of ACP forms an H-bond with the carboxyl oxygen of Asp91, which coordinates the Mg^2+^ ion (Figure 1D).

As observed in other crystal structures of spider venom PLDs, the Mg^2+^ ion is stabilized at the active site through three coordination bonds with the carboxyl oxygens of Glu32, Asp34, and Asp91 (Figure 1C,D) [28]. A water molecule provides the fourth coordination bond with Mg^2+^ (Figure 1C,D). As mentioned earlier, ACP coordinates with the metal cofactor via two of its phosphate oxygens, one of which is part of the ligand’s six-membered ring (Figure 1C,D). Together, these interactions complete the typical sixfold coordination of the Mg^2+^ ion [28].

### 2.2. Formation of ACP at the Active Site

**Figure 2:**
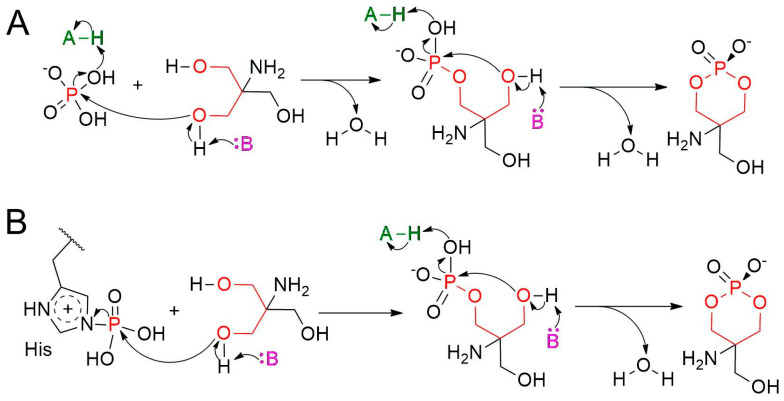
Proposed mechanisms for the formation of ACP catalyzed by *L. intermedia* PLD. (**A**) This mechanism requires the presence of a phosphate ion and Tris bound to the active site. The two precursors are converted into ACP in two steps, involving the phosphorylation of Tris to yield tromethamine phosphate (TMP), followed by cyclization to form ACP, with the concomitant release of two water molecules. (**B**) This mechanism requires the phosphorylation of a PLD catalytic His (see PDB 1V0U), and the binding of Tris. The ACP formation occurs through two sequential phosphorylation reactions, during which His is first displaced by the attacking -OH group of Tris, followed by the cyclization of TMP, as shown in (**A**). Species A (green) and B (purple) represent general acids and bases, respectively. Residues His12 and His47 have been proposed to play the roles of species A and B during headgroup cleavage in phospholipid substrates [19]. One of these His residues must be phosphorylated in mechanism B. Atoms forming the six-membered ring are colored red.

We formulated two plausible hypotheses, both involving two sequential phosphorylation steps, to account for ACP formation by *L. intermedia* PLD (Figure 2). In the first mechanism, phosphate ions from the solution, likely coordinated to the Mg^2+^ cofactor, interact with Tris. These precursors originate from the purification and crystallization buffers, respectively [30]. ACP then forms through the sequential nucleophilic attacks by two Tris hydroxyl groups on the phosphate P atom, with the concomitant release of two water molecules (Figure 2A). A second mechanism differs in the source of the phosphate moiety in ACP (Figure 2B). This alternative mechanism is based on evidence showing that a catalytic histidine in a *Streptomyces* PLD can undergo phosphorylation (PDB 1V0U) [25]. In this enzyme, phosphohistidine formation has been attributed to the double catalytic processing of phospholipids [25]. We hypothesize that during expression and lysis, *L. intermedia* PLD may interact with cellular components, leading to the phosphorylation of one of its two catalytic histidines (His12 or His47). In this mechanistic model, the first step involves the displacement of the His residue through a nucleophilic attack by a Tris hydroxyl group on the P atom, while the second step is identical to that of the first mechanism (compare the second steps in Figure 2A,B).

The binding of Tris to the PLD active site is a critical step for both mechanisms. To address the feasibility of this process, we conducted two replicate 500 ns MD simulations of *L. intermedia* PLD in complex with phosphate and Tris, followed by thermodynamic integration (TI) free energy calculations. The initial conformation for the simulation was derived from the reprocessed enzyme’s crystal structure, where the crystallographic ligand was manually modified to generate a phosphate and a Tris-bound molecule.

**Figure 3:**
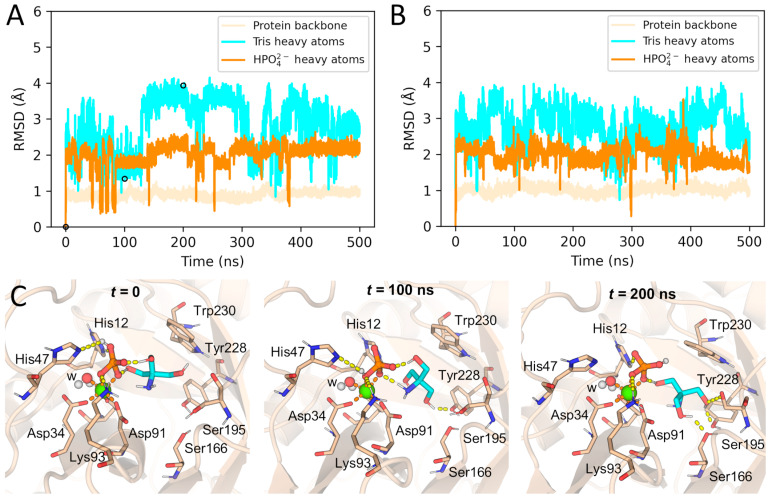
Results of the MD simulation of *L. intermedia* PLD in complex with HPO_4_^2−^ and Tris. (**A**,**B**) RMSD plots calculated for the groups of atoms indicated in the legends, using the initial structure (*t* = 0) as a reference and during two replicate 500 ns MD simulations. All frames were aligned to the protein’s backbone in the initial structure before the RMSD calculations. (**C**) Structural representations of the active site and the bound molecules of HPO_4_^2−^ and Tris in three snapshots, extracted from the first replicate MD simulation at *t* = 0, 100, and 200 ns, which correspond to distinct regions in the RMSD plot (black circles in (**A**)). H-bonds and coordination bonds are shown as yellow and orange dashed lines, respectively. The Mg^2+^ ion, the P atom of HPO_4_^2−^, and the C atoms of Tris are represented in green, orange, and cyan, respectively. A water molecule coordinating Mg^2+^ was included in all structural representations.

Root-mean-square deviation (RMSD) analysis of the two ligands throughout the replicate MD simulations revealed that both phosphate and Tris remained stably bound to the active site, with the maximum RMSD values slightly exceeding 4 Å (Figure 3A,B). Structural snapshots taken from the first replicate MD simulation at *t* = 0, 100, and 200 ns, which are representative of different regions in the RMSD plots, further support this observation (Figure 3C). Notably, the Tris binding mode sampled at *t* = 200 ns seems to be stabilized by the formation of additional H-bonds with active-site residues when compared to the other two snapshots (Figure 3C). Alchemical binding free energy calculations, initiated with the 200 ns snapshot (Figure 3C), estimated the affinity of Tris for the PLD-phosphate complex to be −4.07 kcal/mol (Table S1, Supporting Information). This negative value indicates the spontaneous binding of Tris to the protein’s active site in the analyzed conformation. This result is also in agreement with the stability observed during the two replicate MD simulations.

The formation of tromethamine phosphate (TMP, PubChem CID 19848425) through the mechanism in Figure 2A was analyzed from the replicate 500 ns MD simulations of *L. intermedia* PLD in complex with phosphate and Tris. Although the pK_a_ of Tris (~8.1) indicates that its amine nitrogen is protonated at the crystallization pH (7.5) [30], we modeled it as deprotonated, a state that could be feasibly favored by the chemical environment of the active site. This protonation choice allowed us to assess the relative nucleophilic potential of the Tris hydroxyl groups compared to its deprotonated N atom by measuring their distances to the phosphate’s P atom throughout the MD simulations. As shown in Figure S2A,C (Supporting Information), the hydroxyl O atoms of Tris were consistently positioned closer to the P atom than the N atom, with this tendency becoming more pronounced during the final 50 ns of both simulations. Moreover, the 200 ns snapshot (Figure 3C) suggests that the positively charged His12 residue may facilitate the reaction by protonating a leaving oxygen atom of phosphate, thereby promoting its release during the nucleophilic attack by the closely positioned Tris hydroxyl group.

After the previous analyses, an important question remained regarding the identity of the base (B) responsible for abstracting the proton from the hydroxyl group of Tris during the first step (Figure 2A). Structural representations of the trajectory frames reveal that residue His47 was positioned distantly from the modeled bound conformation of Tris, and, therefore, it could not participate in proton abstraction. Consequently, we investigated whether a water molecule could serve as the base in this step. To evaluate this possibility, we monitored the distance between the hydroxyl O atom of Tris closest to the phosphate’s P atom and the water molecule closest to this O atom throughout the replicate MD trajectories (Figure S2B,D, Supporting Information). The analysis revealed that water molecules were consistently positioned at distances ranging from 2.5 to 5 Å from the attacking hydroxyl O, supporting their potential role as a base in the reaction. Alternatively, given Tris’s small size, it may adopt multiple conformations within the PLD active site, some of which could potentially bring it into closer proximity to His47. However, such conformations were not sampled during the MD simulations performed here, which were initiated from a bound Tris conformation similar to that of ACP in the crystal structure.

The second proposed mechanism for ACP formation (Figure 2B) involves the prior phosphorylation of a catalytic His residue through phospholipid processing. In the following section, we explore whether the catalytic His in *L. intermedia* PLD can serve as a nucleophile to attack the substrate’s P atom. Both mechanisms in Figure 2 involve ACP formation via an intramolecular nucleophilic attack by the TMP hydroxyl oxygen on the P atom, a process resembling the PLD-catalyzed phospholipid cyclization. Our investigation into the nucleophilic potential of the hydroxyl group will shed light on this final step. To ensure biological relevance, we selected SM, the natural substrate, for our computational study.

### 2.3. QM/MM Calculations Support the Non-Covalent Mechanism

**Figure 4:**
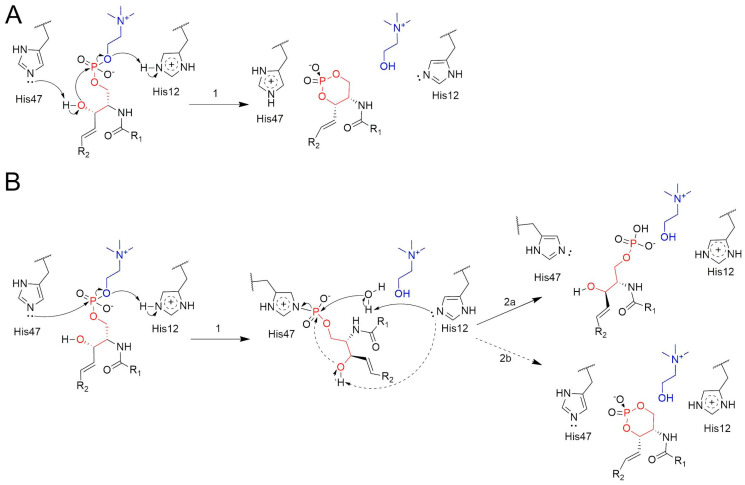
Proposed mechanisms for SM cleavage by spider venom PLDs. (**A**) Non-covalent mechanism initiated by the intramolecular nucleophilic attack of the SM free hydroxyl group on the P atom, yielding the cyclic phosphate CC(1,3)P. (**B**) Covalent mechanism initiated by the nucleophilic attack of the NE2 atom of His47 to the SM P atom. This mechanism has been proposed to yield linear phosphate products (step 2a), as a consequence of the nucleophilic attack by a water molecule on the P atom, which results in the release of the His moiety (see solid arrows). Alternatively, the covalent mechanism may be compatible with the formation of cyclic phosphates (step 2b) if the SM hydroxyl group carries out the attack on the P atom, displacing the His moiety (see dashed arrows). The atoms forming the six-membered ring are colored red, while those belonging to the headgroup are colored blue.

We evaluated the initial step of two alternative mechanisms underlying the cleavage of the SM headgroup by spider venom PLDs. The first, a non-covalent mechanism, involves an intramolecular attack by the hydroxyl group of SM on its P atom, with the catalytic His residues facilitating the acid–base catalysis (Figure 4A) [19,28]. The second, a covalent mechanism, was originally proposed by Leiros et al. for a *Streptomyces* PLD [25], and later postulated by Murakami et al. for spider venom PLDs [14,20]. This mechanism begins with a nucleophilic attack by a catalytic histidine on the P atom [14,20,25,28], leading to the formation of a phosphohistidine intermediate (step 1, Figure 4B). While this mechanism has been associated with the generation of linear phosphate products (step 2a, Figure 4B), it does not necessarily exclude the formation of cyclic phosphates, which are the products of spider venom PLDs [26]. Indeed, following phosphohistidine formation, the N–P bond may be cleaved either by hydrolysis (step 2a, Figure 4B) or via an intramolecular attack by the substrate’s hydroxyl oxygen (step 2b, Figure 4B).

Furthermore, the mechanisms proposed in Figure 4 assign specific roles to each catalytic His residue, which are based on previous structural findings describing SM accommodation within the PLD active site. In fact, the modeled PLD–substrate structures indicate that His12 is positioned closer to the oxygen atom of the leaving group (choline), while His47 can interact with either the attacking hydroxyl group or the P atom [28,29]. This analysis confirms the roles attributed by Murakami et al. to the His residues in the revised version of the covalent mechanism [14].

Free energy profiles were generated for the initial step of each mechanism (step 1 in Figure 4A,B) using a combination of QM/MM and umbrella sampling (US) techniques. The results allowed us to evaluate whether the nucleophilic attack on the P atom was performed by the substrate’s hydroxyl oxygen (non-covalent mechanism) or by the NE2 atom of His47 (covalent mechanism). Figure S3 (Supporting Information) presents the optimized initial configuration, indicating the key amino acid residues and relevant interatomic distances.

**Figure 5:**
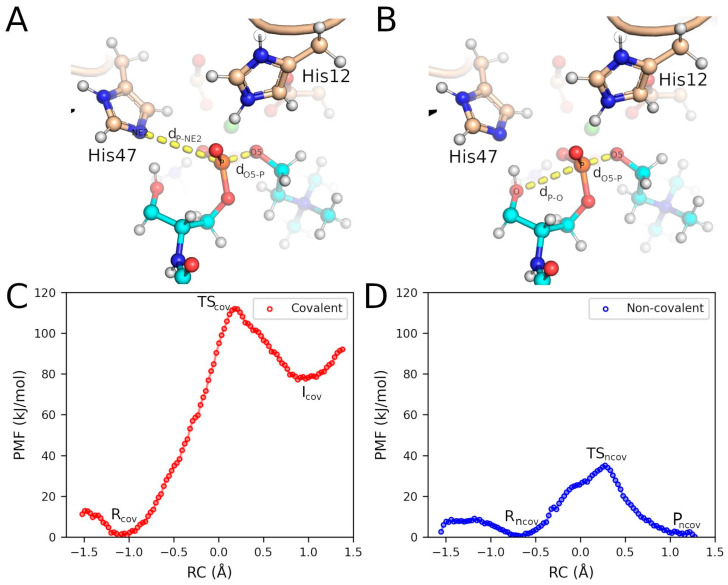
Energy profiles obtained for the two mechanisms. (**A**,**B**) The interatomic distances (dashed lines) used to define the reaction coordinates (RCs) for the covalent and non-covalent mechanisms, respectively. (**C**,**D**) The PMFs obtained along the chosen RCs for the two mechanisms. R, TS, I, and P stand for the initial state, the transition state, the intermediate, and the products, respectively, with subindices *cov* and *ncov* denoting whether they correspond to the covalent and non-covalent mechanisms.

**Figure 6:**
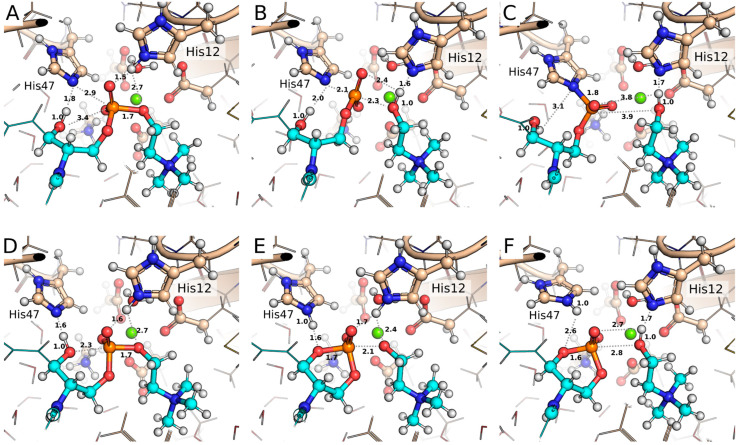
Structural representations of key snapshots obtained from US simulations. (**A**–**C**) Covalent mechanism: (**A**) initial configuration (R), (**B**) transition state (TS_cov_), and (**C**) covalent intermediate (I_cov_). (**D**–**F**) Non-covalent mechanism: (**D**) initial reorganized configuration (R_ncov_), (**E**) transition state (TS_ncov_), and (**F**) product (P_ncov_). Atoms depicted as lines correspond to the MM region, while those shown as balls and sticks represent the QM region. Carbon atoms from the protein and substrate (SM) are colored soft brown and cyan, respectively. Relevant distances are shown in Å.

The potential of mean force (PMF) for the covalent mechanism reveals an energy barrier of 112.0 kJ/mol for the initial step, leading to the formation of the covalent intermediate with the enzyme (I_cov_) (Figure 5A,C and Figure S4A, Supporting Information). In the initial configuration (R), the NE2 atom of His47 is partially oriented toward the P atom of the substrate (SM), a position that is facilitated by an H-bond between NE2 and the hydroxyl group of SM (O-H) (Figure 6A). The formation of the transition state involves a spontaneous proton transfer from HE2 (His12) to O5, which facilitates the headgroup release (Figure 6B). After the nucleophilic attack, the resulting covalent intermediate structure (I_cov_) shows the P atom bonded to NE2 (His47) and the newly formed choline molecule (Figure 6C).

The free energy profile for the non-covalent mechanism initially shows a minor barrier of 6.2 kJ/mol, associated with the rotation of the O-H of SM, which, while maintaining the H-bond with NE2, favorably reorients the oxygen atom toward the P atom (Figure 5B,D, Figure S4B, Supporting Information). This configuration is designated as R_ncov_ (Figure 6D), and its energy difference relative to conformation R is −3.1 kJ/mol. The transition state (TS_ncov_) exhibits an energy barrier of 35.1 kJ/mol, characterized by the complete transfer of the proton from the hydroxyl group to NE2 (His47) and the formation of a regular pentacoordinate phosphate, with an angle of 171.5° between O (SM), P (SM), and O5 (SM) (Figure 6E). The proton transfer from HE2 (His12) to O5 (SM) occurs spontaneously immediately after the transition state formation. The final configuration, P_ncov_, is more stable than the initial configurations, resulting in the formation of cyclic phosphate and choline as products (Figure 6F).

## 3. Discussion

The re-analysis of the crystal structure of *L*. *intermedia* PLD (PDB: 3RLH) revealed the presence of a novel cyclic phosphate (ACP) bound at the enzyme’s active site. This finding aligns with previous studies proposing that spider venom PLDs catalyze transphosphatidylation reactions, leading to the formation of cyclic products [19,26]. The identified cyclic phosphate establishes specific interactions with the catalytic residues His12 and His47, as well as the Mg^2+^ cofactor, highlighting their role in stabilizing the reaction intermediate and ensuring substrate specificity.

We propose two hypotheses for the formation of ACP at the active site of *L. intermedia* PLD. The first hypothesis involves the coordination of phosphate to the Mg^2+^ cofactor, followed by the binding of Tris to the enzyme’s active site. The binding free energy calculations indicate that Tris binding occurs spontaneously. We suggest that the enzyme catalyzes the phosphorylation of Tris through a two-step process, with the second step involving TMP cyclization to form ACP. These steps closely resemble the non-covalent mechanism proposed for PLDs, with the key difference being that water, rather than a primary alcohol, is the leaving group during the substitution reactions. Previous findings have shown that small primary alcohols may serve as nucleophiles during the transphosphatidylation reactions catalyzed by PLDs [1], thus providing support for the role of Tris in the proposed mechanism of ACP formation. The second hypothesis for ACP formation, involving the participation of a phosphohistidine residue, is not supported based on our QM/MM results assessing the ability of the catalytic His to perform a nucleophilic attack on the phosphate P atom, as discussed below.

This study concludes with a computational analysis of two alternative mechanisms—covalent and non-covalent—for the cleavage of the phospholipid headgroup by a spider venom PLD. The covalent mechanism involves the formation of an His47-SM adduct, with His12 facilitating the headgroup release via protonation of the leaving O atom. This mechanism is well-documented for a *Streptomyces* PLD [25]. In contrast, the non-covalent mechanism entails the formation of a cyclic phosphate via an intramolecular attack by the hydroxyl (-OH) group of SM on the P atom. His47 facilitates deprotonation of the attacking -OH group, while His12 protonates the leaving headgroup [19,28,31]. Although previous studies have shown that spider venom PLDs lead to the formation of cyclic phosphates [19,26], the precise identity of the nucleophile in the initial catalytic step remains unresolved, as either a noncovalent or a modified covalent mechanism could account for these products.

The initial step of each mechanism was successfully modeled, and our calculations confirm the crucial role of the substrate’s hydroxyl group in both cases. In the covalent mechanism, it helps correctly orient His47, while in the non-covalent mechanism, it facilitates the intramolecular nucleophilic attack and the formation of the cyclic phosphate product. This could explain why a *Bacillus cereus* SMase does not cleave a 3O-methylated SM derivative [32].

The energy barrier for the initial step of the non-covalent mechanism was estimated to be 35 kJ/mol—approximately 77 kJ/mol lower than that of the initial step of the covalent mechanism. This significant energy difference strongly favors the non-covalent pathway. Thus, our results support the substrate’s hydroxyl group as the nucleophile driving headgroup release via an intramolecular transphosphatidylation reaction, as previously suggested [19,26,31]. Furthermore, this conclusion, combined with the established role of His as a nucleophile in other PLDs [25], suggests that not all members of the PLD superfamily share the same mechanism.

Overall, our findings provide new structural and energetic evidence supporting the non-covalent mechanism. Based on the available experimental data, we propose a mechanism in which His47 deprotonates the substrate’s hydroxyl group, enabling the negatively charged oxygen to perform an intramolecular nucleophilic attack on the P atom. This step triggers the release of the polar headgroup (choline). His12 is proposed to protonate the oxygen atom of choline, thereby enhancing its potential as a leaving group. Additionally, we suggest that water molecules mediate the protonation of His12, the deprotonation of His47, and the dissociation of CC(1,3)P from Mg^2+^, thus regenerating the active site for subsequent catalytic cycles [28]. Structural analysis further indicates that product dissociation follows a specific order, with CC(1,3)P being released first to clear the pathway for the more deeply buried choline.

The present refined structure, with a cyclic phosphate (ACP) bound at the active site, serves as an avenue for the design of PLD inhibitors. In this context, several computational techniques offer promising avenues for inhibitor development. The ACP molecule constitutes a valuable query to identify potential new ligands through similarity searches in chemical libraries. The complex structure can also be leveraged to generate pharmacophore models, further aiding in the identification of novel PLD inhibitors.

## 4. Conclusions

In this study, we reanalyzed the crystal structure of *L. intermedia* PLD and identified the presence of cyclic phosphate ACP at the enzyme’s active site. This finding provides new evidence supporting the formation of cyclic products by spider venom PLDs. Furthermore, using QM/MM simulations, we examined the initial step of two proposed catalytic mechanisms—covalent and noncovalent. Our results indicate that the noncovalent mechanism, in which the SM hydroxyl oxygen undergoes an intramolecular attack on the P atom, is strongly favored over the covalent mechanism, which involves the formation of a His-SM adduct. These insights not only enhance our understanding of PLD catalysis but may also aid in the design of novel inhibitors targeting these enzymes.

## 5. Materials and Methods

### 5.1. Refinement of the PDB 3RLH Crystal Structure

The crystal structure of the PLD from *L*. *intermedia* (PDB ID: 3RLH) [30] was revisited using PyMOL [33], CCP4 [34], and WinCoot [35]. Two PEG4 molecules (Figure S1, Supporting Information), which were initially assigned to electron densities near the active site residues (His47 and His12), the ion-binding site (Glu32, Asp34, and Asp91), and the Mg^2+^ cofactor, were removed. Subsequently, other potential ligands were manually inserted into these positions using WinCoot [35]. Following this step, refinement cycles were performed using REFMAC5 [34], and the stereochemical parameters were evaluated using the MolProbity server [36]. The new coordinates, including (5-amino-2-hydroxy-2-oxo-1,3,2λ^5^-dioxaphosphinan-5-yl)methanol (abbreviated as ACP) (Figure S1, Supporting Information), replaced the coordinates in PDB 3RLH previously deposited by Giuseppe et al. [30].

### 5.2. Ligand Parametrization for MD Simulations

Quantum mechanical (QM) calculations were performed using Gaussian 09 [37] and following the protocol described by Hernandez-Gonzalez JE et al. to calculate the electrostatic potential (EP) of several small ligands studied in this work [38]. The resulting EP was used to derive partial atomic charges via the RESP method, as implemented in Antechamber [39]. Bonded and nonbonded parameters were assigned with the *parmchk2* program using the GAFF2 force field [39].

### 5.3. MD Simulation Setup

The program *tleap* from Amber22 [39] was used to obtain the topology and coordinates files necessary for running MD simulations of the *L. intermedia* PLD (PDB ID 3RLH) in complex with several small ligands. The protein was parameterized with the ff19SB force field [40], with the ligands being parametrized as explained in the previous section. OPC water molecules were added to an octahedral simulation box, whose edges spanned 10 Å from the solute surface. Cl^−^ counterions were added to neutralize the net charge of the simulation box. The system was subjected to energy minimization, followed by heating in the NVT ensemble and equilibration in the NPT ensemble, both conducted for 500 ps with harmonic restraints applied to the heavy atoms of the protein, the ligand, and the cofactor (k = 10 kcal·mol^−1^·Å^−2^). During heating, the temperature was increased from 10 to 300 K using a linear gradient, with the system coupled to a Berendsen thermostat. Random initial velocities drawn from a Maxwell–Boltzmann distribution at the initial temperature were assigned to the system’s atoms. The NPT equilibration was carried out at pressure 1 bar and temperature 300 K. The pressure was controlled by means of the Berendsen barostat. Finally, 500 ns productive NVT MD simulation was performed in the presence of mild harmonic restraints (k = 2 kcal·mol^−1^·Å^−2^) applied to the heavy atoms of residues Glu32, Asp34, Asp91, and Mg^2+^ to preserve the geometry of the coordination bonds. A replicate 500 ns MD simulation under the same conditions was performed by assigning different random velocities to the system’s atoms during the heating phase. All the previous steps were carried out with pmemd.cuda of Amber22 [39]. Long-range electrostatic interactions were handled with the particle mesh Ewald (PME) algorithm. A timestep of 2 fs was used in all simulations, with SHAKE applied to constrain the bond lengths of hydrogen atoms to their corresponding heavy atoms. During the production runs, the temperature was controlled with the Langevin thermostat [39].

### 5.4. Alchemical Free Energy Calculations

TI was employed to estimate the absolute binding free energy of tris(hydroxymethyl)aminomethane (Tris) to the PLD active site. The ligand, subjected to the parametrization protocol presented before, was superimposed on the equivalent atoms of the cyclic phosphate placed in the newly refined crystal structure. A phosphate ion coordinating the Mg^2+^ cofactor was also included in the complex structure. The program *tleap* [39] was used to obtain the topology and coordinate files of the PLD bound to phosphate and Tris and of the latter molecule free in the water. Distance, angular, and dihedral restraints involving three heavy atoms of Tris and three Cα atoms of the protein were set in order to prevent the ligand from escaping the active site during the alchemical decoupling, according to the protocol proposed by Boresch et al. [41]. A two-step scheme, where the electrostatic and van der Waals transformations are conducted independently, was followed. Each of the four transformations, i.e., ligand discharging at the active site and being free in water, and the decoupling of the van der Waals parameters of the ligand in the same two states, was carried out through 21 hybrid states, corresponding to λ values 0, 0.05, …, 0.95, 1.

Each hybrid state was subjected to energy minimization, 500 ps NVT heating, 10 ps NPT equilibration, and 10 ns production runs. The conditions were similar to those used for the conventional MD simulations. Soft-core potentials were set during the van der Waals transformation of the ligand. All the steps were carried out with pmemd.cuda of Amber22 [39]. The collected values of the derivative of the potential energy with respect to λ for all hybrid states in each alchemical transformation were used to estimate the associated free energies via integration. The alchemical analysis.py program was employed to calculate the alchemical free energies and the associated errors [42]. A 10 ns conventional unrestrained MD simulation was conducted to account for the impact of removing the Boresch restraints from the ligand using a single-step FEP equation [43]. Moreover, the analytical expression derived by Boresch et al. was used to determine the free energy associated with restraining the decoupled ligand to the active site [41].

### 5.5. QM/MM Simulations

The last snapshot from the MD simulation of the *L. intermedia* PLD in complex with SM was used as the starting point for the QM/MM potential calculations [29]. All QM/MM simulations were performed using pDynamo 3 [44], coupled with xTB version 6.6.0. The EasyHybrid3 graphical [45] was utilized for setting up and executing all hybrid simulations. The subset used for the QM/MM calculations consisted of 9549 atoms within a sphere of 27 Å radius, centered on the substrate’s P atom. To maintain the structural integrity of the system, the positions of atoms beyond 25 Å from the sphere center were constrained during the sampling calculations. The system was divided into QM and MM regions, as illustrated (Figure S3, Supporting Information). The QM region included 92 atoms (expanded to 100 with boundary-saturating hydrogens) and carried a total charge of +1. This region comprised the side chains of His12, Glu32, Asp34, Hid47, Asp91, the Mg^2+^ ion, a water molecule, and 37 atoms from the substrate, including the choline group. The pDynamo hydrogen link atom approximation was used to saturate bonds at the QM/MM boundary. Hybrid potentials were employed, combining GFN2-xTB [46] for the QM region and Amber ff14SB [47] for the MM region. The system was relaxed through geometry optimization using the conjugate gradient algorithm, with a convergence criterion of an RMSD of 0.1 Å on the atomic positions.

The structures of the reaction products for two distinct mechanisms were sampled through relaxed reaction coordinate scans. These scans were conducted with 0.1 Å intervals between the steps, applying a harmonic restraining potential of 4000 N/mol to the reaction coordinate. For the covalent mechanism, the chosen reaction coordinate (RC_cov_) was defined by two interatomic distances: d_O5–P_ (between O5 and P from the substrate) and d_P–NE2_ (between P from the substrate and NE2 from His47) (Figure 5A). For the non-covalent mechanism, the reaction coordinate (RC_ncov_) was defined by d_O5–P_ and d_P–O_ (between P and O atoms from the substrate) (Figure 5B). Each step in the scan was relaxed with 6000 iterations using the conjugate gradient algorithm. These potential energy scans of the reaction coordinates were complemented with calculations to determine the free energy profiles along the reaction coordinate using US combined with the weighted histogram analysis method (WHAM). For the covalent and non-covalent mechanisms, 47 and 43 US windows were employed, respectively. In both cases, the velocity Verlet integration algorithm was applied. Each window involved 20 ps of equilibration dynamics followed by 50 ps of production dynamics, using distance-dependent harmonic biasing potentials with force constants of 2700 kJ·mol^−1^·Å^−2^. The WHAM analysis utilized 100 bins, a maximum of 1000 iterations, an RMS gradient tolerance of 1.0 × 10^−3^·kJ·mol^−1^, and a temperature of 300 K.

## Figures and Tables

**Table 1:** Structure refinement statistics for deposited structures (3RLH) and reprocessed data.

Refinement	3RLH	3RLH (Reprocessed)
R_factor_	17.2	16.7
R_free_	21.4	19.7
RMSD bond distances (Å)	0.023	0.0185
RMSD bond angles (°)	1.930	1.57
Ramachandran outliers (%)	0	0
Ramachandran favored (%)	99.3	98.9
Waters molecules	184	190
Ligands	Mg^2+^, PGE ^a^, PEG ^b^ and EDO ^c^	Mg^2+^, PGE ^a^, PEG ^b^, EDO ^c^ and ACP ^d^

^a^ Monoethylene glycol; ^b^ polyethylene glycol; ^c^ ethylene glycol; ^d^ (5-amino-2-hydroxy-2-oxo-1,3,2λ^5^-dioxaphosphinan-5-yl)methanol.

## Data Availability

The original contributions presented in this study are included in the article/Supplementary Materials. Further inquiries can be directed to the corresponding author.

## Supplementary Materials

The following supporting information can be downloaded at: https://www.mdpi.com/article/10.3390/toxins17030111/s1, Figure S1: Ligands identified in the crystal structure of PLD from *Loxosceles intermedia* (PDB ID: 3RLH); Figure S2: Interatomic distance profiles during the 200 ns MD simulation of *L. intermedia* PLD bound to Tris and phosphate; Figure S3: Representation of the hybrid QM/MM system; Figure S4: Histograms obtained for every US window along the reaction coordinate; Table S1: Absolute binding free energy calculation for the PLD-Tris complex in the presence of HPO_4_^2−^ coordinating the Mg^2+^ cofactor.
