# Supplementary material for: Structural and Energetic Evidence Supports the Non-Covalent Phosphate Cyclization by the Class II Phospholipase D from Loxosceles intermedia
Source: Toxins (Basel). 2025 Feb 27;17(3):111. doi: 10.3390/toxins17030111 (PMC11945750; doi:10.3390/toxins17030111)
Supplement: Supplementary file 1 [file toxins-17-00111-s001.zip › toxins-3471432-supplementary.pdf]

Supplementary Materials: Structural and Energetic Evidence Supports the Non-Covalent Phosphate Cyclization by the Class II Phospholipase D from *Loxosceles intermedia*

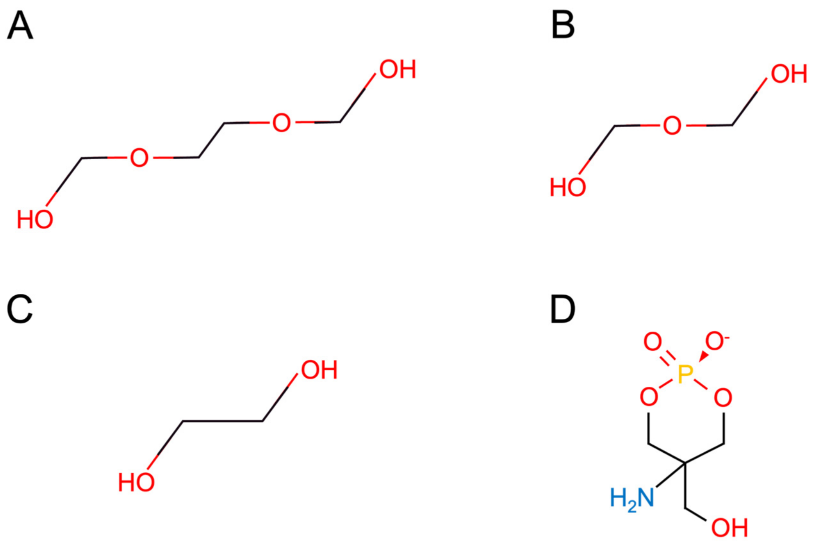

Figure S1. Ligands identified in the crystal structure of PLD from *Loxosceles intermedia* (PDB ID: 3RLH). A) Monoethylene glycol, B) Polyethylene glycol, C) Ethylene glycol, and D) (5-amino-2-hydroxy-2-oxo-1,3 ,2λ⁵-dioxaphosphinan-5-yl)methanol (ACP).

Table S1. Absolute binding free energy calculation for the PLD-Tris complex in the presence of HPO_4^{2-} coordinating the Mg^{2+} cofactor

System	ΔG_{dech} [a] (kcal/mol)	ΔG_{vdw} [b] (kcal/mol)	ΔG^{dum}_{rest} [c] (kcal/mol)	ΔG^{int}_{rest} [d] (kcal/mol)	ΔG_{sym} [e] (kcal/mol)	ΔG^{o}_{bind} (kcal/mol)
PLD/Mg^{2+}/HPO_4^{2-} -Tris	1.42 (0.20)	-8.11 (0.22)	6.5	-3.18 (0.51)	-0.65	-4.07 (0.59)

a. The free energy variation associated with decharging the ligand in solution and when bound to the protein, calculated as: $\Delta G_{dech}=\Delta G_{solv,dech}-\Delta G_{prot,dech}$

b. The free energy variation related to the disappearance of the decharged ligand's van der Waals interactions in solution and when bound to the protein, calculated as: $\Delta G_{vdw}=\Delta G_{solv,vdw}-\Delta G_{prot,vdw}$

c. Free energy variation stemming from the addition of restraints to position the non-interacting ligand at the binding site, including a correction for the molar standard state based on a defined volume of 1661 $Å^3$.

d. Free energy variation associated with the removal of the same restraints after the ligand becomes fully interacting at the protein's binding site, determined using TI.

e. Free energy variation associated with broken symmetry due the addition of restraints. The Tris molecule has three chemically identical hydroxymethyl groups bound to the same carbon, thus ΔG_{sym}= -RTln(3).

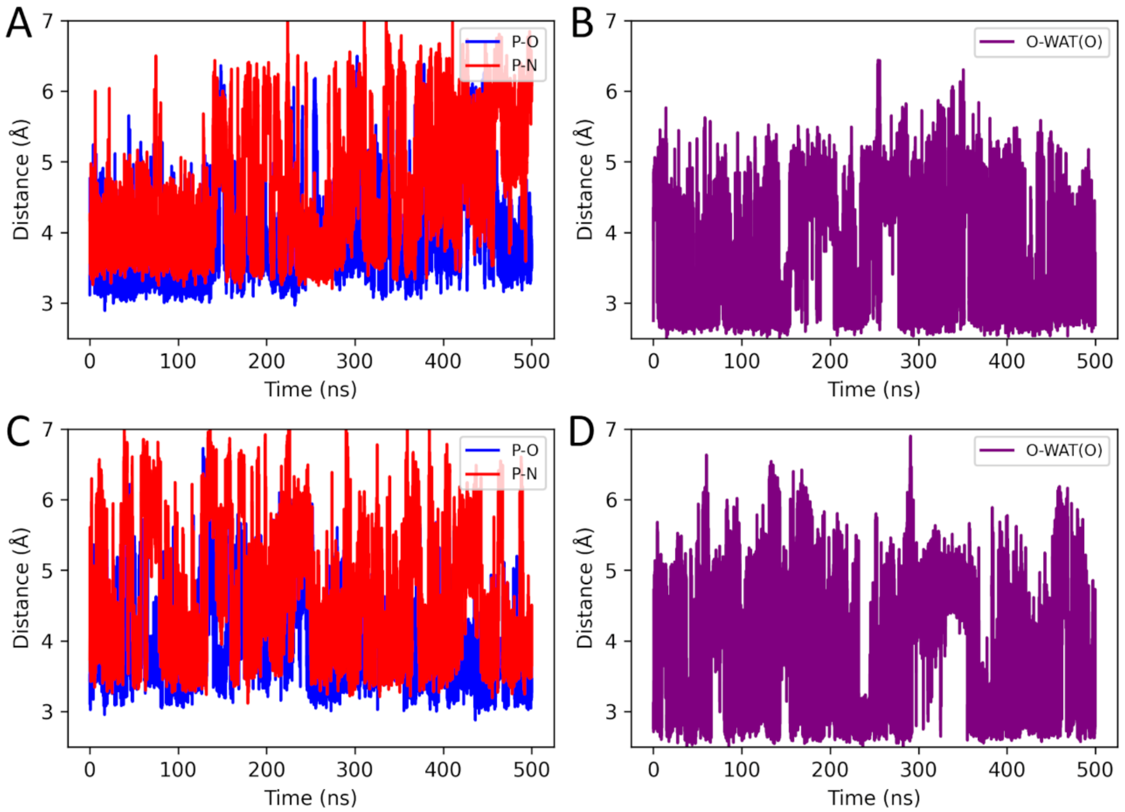

Figure S2. Interatomic distance profiles during the 200 ns MD simulation of *L. intermedia* PLD bound to Tris and phosphate. A) and C) Minimum interatomic distance between the P atom of phosphate and any of the chemically equivalent O atoms of Tris (blue), and the interatomic distance between the P atom of phosphate and the N atom of Tris (red) during the two replicate MD simulations. B) and D) Distance between the O atom of the closest water molecule and the O atom of Tris that is nearest to the P atom of phosphate during the two replicate MD simulations.

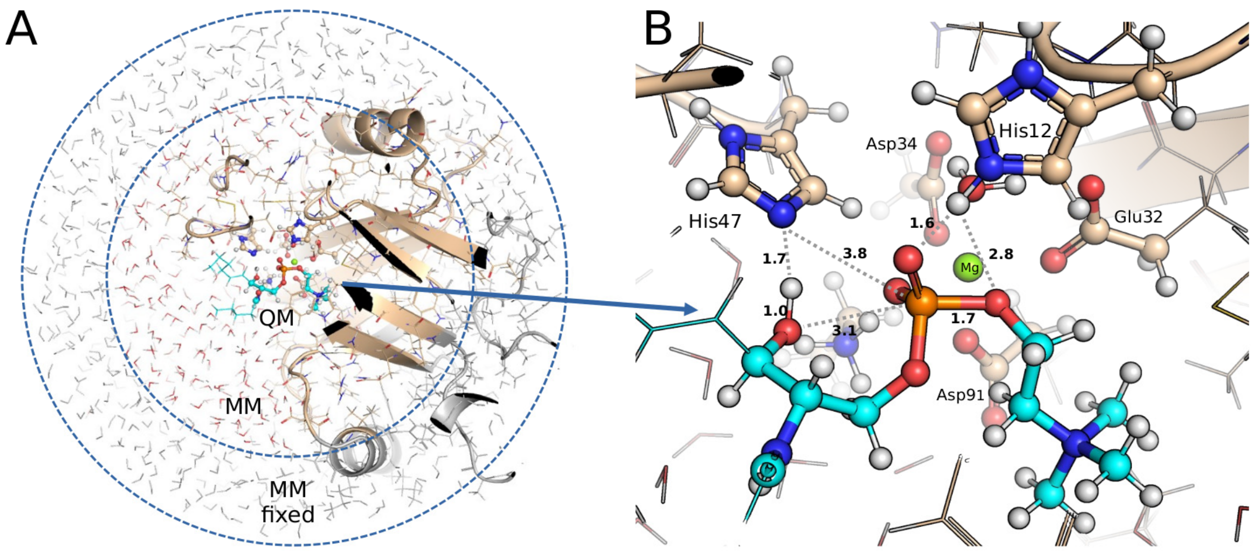

Figure S3. Representation of the hybrid QM/MM system. A) Overall view: atoms depicted as lines are described by molecular mechanics (MM), while atoms shown as ball-and-stick represent the quantum mechanics (QC) region. Fixed atoms in the MM region are shown in gray. B) Initial geometry, including amino acid residues and key interatomic distances, was obtained from geometry optimization. The selected quantum region comprises 92 atoms, expanded to 100 with boundary-saturating hydrogens (not shown).

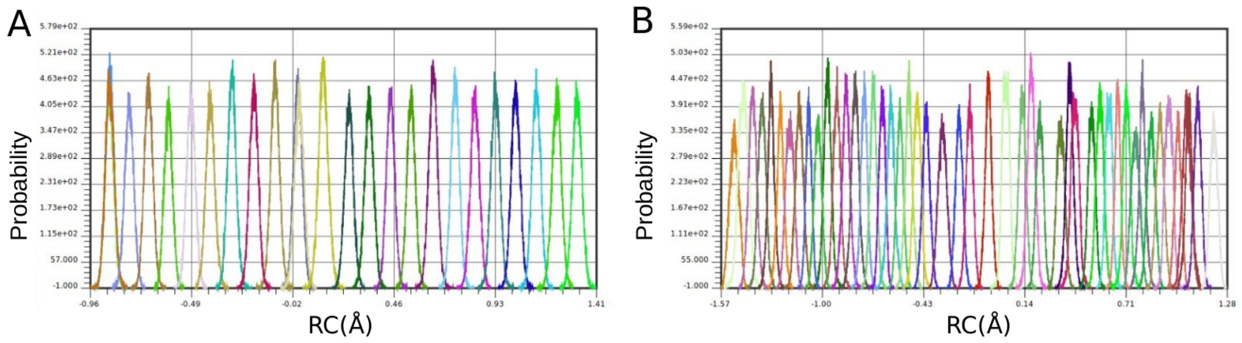

Figure S4: Histograms obtained for every US window along the reaction coordinate. A) covalent and B) non-covalent mechanisms. The overlap of adjacent histogram edges indicates the high quality of the PMF results obtained.
